# Disrupted Lymph Node and Splenic Stroma in Mice with Induced Inflammatory Melanomas Is Associated with Impaired Recruitment of T and Dendritic Cells

**DOI:** 10.1371/journal.pone.0022639

**Published:** 2011-07-21

**Authors:** Saïdi M. Soudja, Sandrine Henri, Marielle Mello, Lionel Chasson, Amandine Mas, Maria Wehbe, Nathalie Auphan-Anezin, Lee Leserman, Benoît Van den Eynde, Anne-Marie Schmitt-Verhulst

**Affiliations:** 1 Centre d'Immunologie de Marseille-Luminy (CIML), Université de la Méditerranée, UMR6546, Marseille, France; 2 INSERM, UMR631, Marseille, France; 3 CNRS, UMR6102, Marseille, France; 4 Ludwig Institute for Cancer Research and de Duve Institute, Université Catholique de Louvain, Brussels, Belgium; University of Frankfurt - University Hospital Frankfurt, Germany

## Abstract

Migration of dendritic cells (DC) from the tumor environment to the T cell cortex in tumor-draining lymph nodes (TDLN) is essential for priming naïve T lymphocytes (TL) to tumor antigen (Ag). We used a mouse model of induced melanoma in which similar oncogenic events generate two phenotypically distinct melanomas to study the influence of tumor-associated inflammation on secondary lymphoid organ (SLO) organization. One tumor promotes inflammatory cytokines, leading to mobilization of immature myeloid cells (iMC) to the tumor and SLO; the other does not.

We report that inflammatory tumors induced alterations of the stromal cell network of SLO, profoundly altering the distribution of TL and the capacity of skin-derived DC and TL to migrate or home to TDLN. These defects, which did not require tumor invasion, correlated with loss of fibroblastic reticular cells in T cell zones and in impaired production of CCL21. Infiltrating iMC accumulated in the TDLN medulla and the splenic red pulp.

We propose that impaired function of the stromal cell network during chronic inflammation induced by some tumors renders spleens non-receptive to TL and TDLN non-receptive to TL and migratory DC, while the entry of iMC into these perturbed SLO is enhanced. This could constitute a mechanism by which inflammatory tumors escape immune control.

If our results apply to inflammatory tumors in general, the demonstration that SLO are poorly receptive to CCR7-dependent migration of skin-derived DC and naïve TL may constitute an obstacle for proposed vaccination or adoptive TL therapies of their hosts.

## Introduction

The generation of immune responses requires interaction of rare antigen (Ag)-specific T lymphocytes (TL) with DC presenting relevant Ag. Interactions occur in secondary lymphoid organs (SLO) and are highly dependent on their architecture [1]. In SLO, T cell zones (T-zones) contain specialized fibroblastic cells [2], the organization of which maximizes the probability that TL encounter the DC presenting the cognate Ag [3]. Stromal cells, including fibroblastic reticular cells (FRC) present in T-zones and follicular dendritic cells (FDC) present in B-zones, secrete chemokines that recruit and organize distinct zones. CCL21/CCL19 recruit CCR7-expressing TL and DC in T-zones, whereas CXCL13 is critical for B-zone formation [2], [4], [5]. Additionally, FRC secrete other factors necessary for the homeostasis of lymphocytes, such as IL-7 [6] and support TL migration in the LN and spleen [7], [8].

Spontaneous or vaccination-induced tumor-specific immune responses do not develop or are insufficient in patients and in experimental animals with advanced cancers. Several possible explanations for this poor reactivity to tumor Ag have been presented. First, tumor Ag may not be adequately presented in the absence of DC-activation signals evolutionarily associated with infectious agents. This leads to TL “ignorance” or tolerance rather than induction of effector functions [9]. Second, tumor development is often associated with inflammation [10], [11], and tumors may secrete tumor derived factors (TDF) that directly impede immune reactions. Some TDF, such as TGFβ, may affect TL differentiation (reviewed in [12]). Others, such as GM-CSF, may alter DC differentiation [13]. Little is known about possible impacts of TDF on SLO architecture and their consequences for anti-tumor responses. Third, immune suppression in cancer has also been associated with the accumulation in blood, lymphoid organs and tumor of immature-type myeloid cells (iMC), also called myeloid suppressor cells (MDSC) (reviewed in [14]). Originating in the bone marrow, these iMC express Gr1 and CD11b in the mouse. Under normal conditions iMC differentiate into DC, macrophages or granulocytes, but their differentiation appears to be blocked by TDF (reviewed in [15]). No studies have as yet addressed the relationship between accumulation of iMC in cancer and SLO structure.

To study the influence of TDF on SLO organization, we used a model of induced melanoma in which similar oncogenic events induce two phenotypically distinct melanomas, both expressing cancer-germline gene *P1A* concomitant with the induction of oncogenesis [16]. One tumor is poorly pigmented (referred to as Amela), promotes high levels of inflammatory cytokines systemically and induces chronic inflammation, leading to an important mobilization of iMC to the tumor and SLO, whereas the other, highly pigmented (referred to as Mela), does not [17]. The immune system of mice with induced slow progressing Mela tumors appeared to be “ignorant” of the tumor but not suppressed, as these mice remained capable of responding to and of rejecting a P1A-expressing transplanted tumor line originating from an induced melanoma. In mice with aggressively progressing induced Amela tumors associated with inflammation, however, the immune system was suppressed and was incapable of rejecting the P1A-expressing transplanted tumor [17].

In this report we provide evidence that tumors associated with inflammation induce alterations of the stromal cell network of SLO. This remodeling during autochthonous tumor development profoundly alters both TL distribution in the spleen and in TDLN and the capacity of skin-derived DC to migrate to TDLN. Consequently, SLO architecture disruption may have an impact on the successful establishment of immunotherapeutic strategies because it impedes TL and DC localization in T-zones. Affecting stromal cell networks in SLO constitutes a new mechanism by which tumors might escape immune control.

## Results

### Impaired recruitment of skin-derived DC in TDLN of mice developing Amela-melanomas

**Figure 1:**
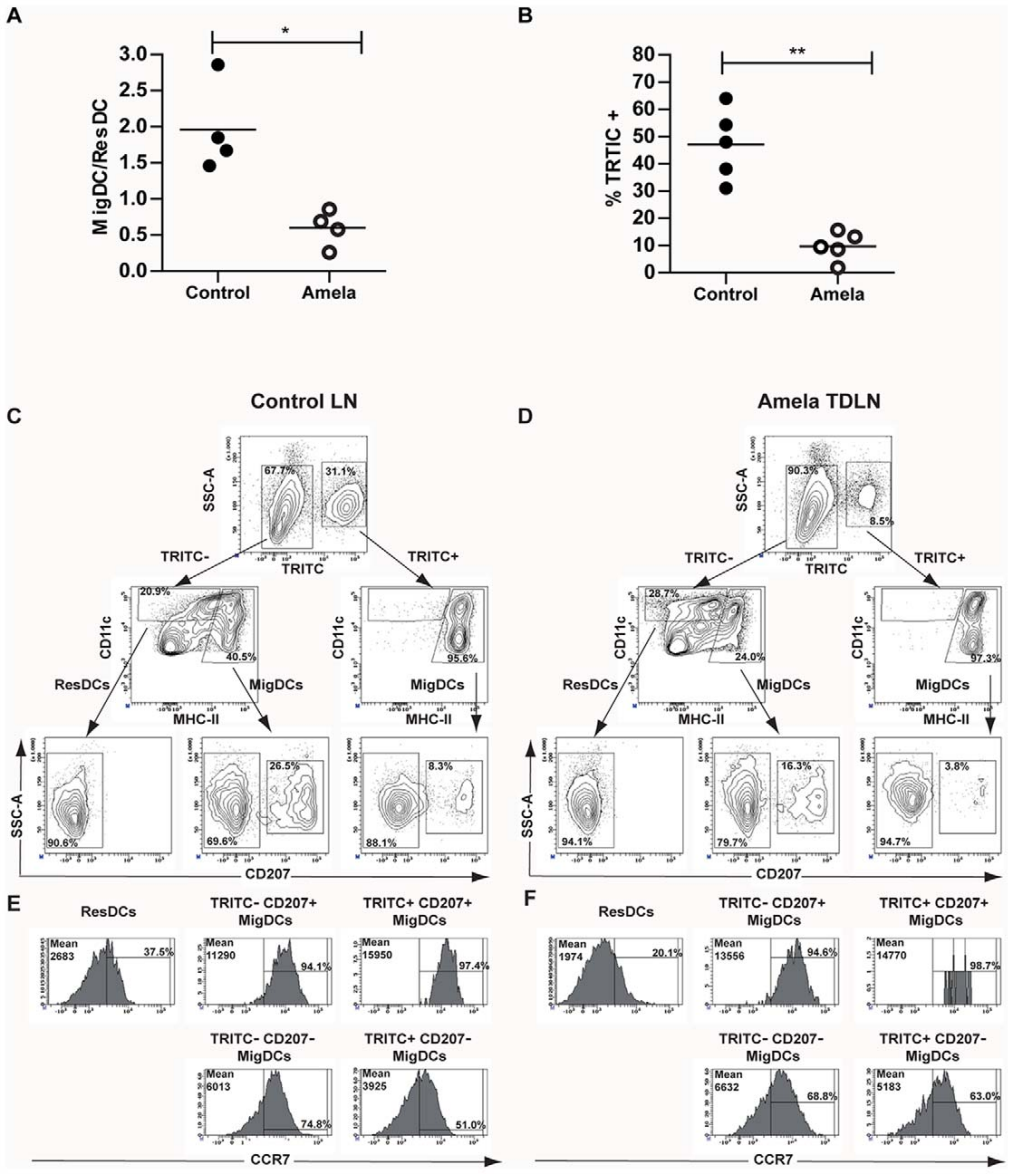
Recruitment of skin-derived DC in mice developing Amela-melanomas is impaired, but their expression of CCR7 is unaltered. (A) Ratio of MigDC versus ResDC in Amela-TDLN or corresponding LN from control mice. (B) % TRITC-labeled cells among MigDC in Amela-TDLN or corresponding LN from control mice 20 hrs after TRITC-skin painting (see Methods). (C-F) Analysis of DC subpopulations in LNs from control (C, E) or Amela-bearing (D, F) mice after TRITC-painting. (C-D) After gating on CD11c^+^NK1.1^−^CD25^−^CD45R^−^ cells, TRITC-staining is shown versus SSC. TRITC^–^ and TRITC^+^ cells were analyzed for expression of CD11c versus MHCII. ResDC and MigDC were found within TRITC^−^ cells; only MigDC were found within TRITC^+^ cells. Only MigDC expressed CD207 corresponding to Langerhans cells and CD207^+^ Dermal DC. (E–F) Histograms of CCR7 expression for the different DC subpopulations indicated in C and D, respectively, show low, intermediate and high fluorescence values (mean), respectively, for ResDC, CD207^−^MigDC and CD207^+^MigDC.

DC take up Ag and migrate from peripheral tissues via afferent lymphatics into T cell areas of draining lymph nodes (DLNs), where they may activate cognate TL. We previously observed that P1A-specific TCRP1A TL [18] adoptively transferred in mice with P1A-expressing induced inflammatory Amela-melanomas were poorly activated in TDLN of these mice [17]. Since TDF can affect DC differentiation [19], we analyzed DC subpopulations present in TDLN of these mice. Skin-DLN contain DC subsets of different origins [20], [21]. These include migratory DC (MigDC), composed of skin-derived epidermal Langerhans cells and dermal DC [22], as well as lymphoid resident DC (ResDC), found in all lymphoid tissues. Cutaneous LN MigDC are CD11c^+^MHCII^high^ while ResDC are CD11c^high^MHCII^intermediate^ ([23], and Fig.1). Using flow cytometry, we observed a significant decrease in MigDC compared to ResDC in TDLN of Amela-bearing mice (Fig.1A). Such a defect was not observed in TDLN from Mela-bearing mice, and was less drastic in contra-lateral as opposed to TDLN from Amela-bearing mice (Fig.S1A-C). By immunohistology (Fig.S1D), MHCII^high^ cells were observed in the T cell zone in control LN and in Mela-TDLN, whereas they were dispersed in Amela-TDLN.

To evaluate the basis for the selective paucity of MigDC in Amela-TDLN, we compared migratory behavior of skin-derived DC from control and tumor-bearing mice. We painted tumor and surrounding skin of mice with induced melanomas or normal skin from control mice with TRITC (see methods). Twenty hours later we analyzed the DC migration into DLN [19]. This showed lower recruitment of TRITC^+^ MigDC in Amela-TDLN than in control LNs (Fig.1B–D).

Migration of DC from the skin to LNs, notably through dermal lymphatics, depends on their expression of CCR7 under both steady state and inflammatory conditions [24]. This was confirmed, in particular for the CD207/langerin^+^ skin-derived DC subpopulation [25]. Deficient CCR7 expression could thus be a cause of defective migration of skin-derived DC to TDLN [26]. To test this possibility, using the TRITC-painting protocol, we compared the level of expression of CCR7 on steady state (TRITC**^−^**) and on recently migrated (TRITC**^+^**) DC subpopulations (Fig.1C–F). As expected, within the TRITC^−^ fraction, we could identify the ResDC as well as the CD207^−^ and CD207^+^ MigDC subpopulations, whereas only the MigDC subpopulations were present in the TRITC^+^ fraction whether in control LN or in TDLN (Fig.1C–D). CCR7 expression was lowest on the ResDC populations and highest on the CD207^+^ MigDC populations in both control LN (Fig.1E) and Amela-TDLN (Fig.1F). The latter expressed similarly high levels of CCR7 in both the TRITC^−^ steady state and the recently migrated TRITC^+^ populations. The CD207^−^ MigDC expressed intermediate CCR7 levels whether in control LN or in TDLN. It should be noted that in the absence of analysis of DC subpopulations, CCR7 expression would have appeared lower in the Amela-TDLN than in control LNs, given that ResDC with low CCR7 expression are enriched relative to MigDC in the Amela-TDLN.

The analysis of CCR7 expression on DC subsets thus revealed that although the MigDC subpopulations were under-represented in the Amela-TDLN, their surface expression of CCR7 was as high as on their counterparts in normal cutaneous LN.

Among other possible causes for poor migration or localization of DC in DLN is the absence of CCR7 ligands, as shown in mice deficient in CCL19 and CCL21-Ser [27]. Such defects would also be expected to affect TL localization in TDLN. This possibility was analyzed next.

### Impaired T cell localization in TDLN of mice developing Amela-melanomas

**Figure 2:**
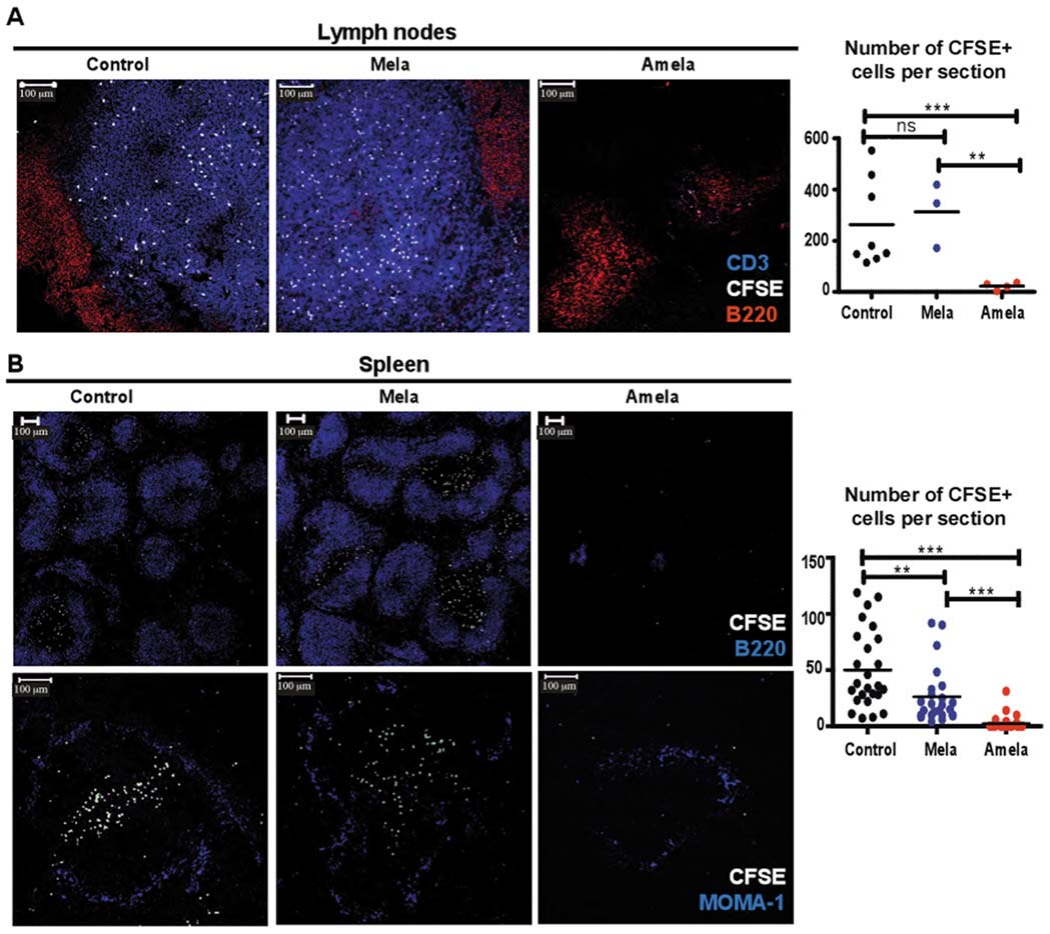
TL localization in TDLN of mice developing Amela-melanomas is impaired. (A) CFSE-labeled CD8 TL from B10.D2 mice (white) were adoptively transferred into control, Mela- and Amela-bearing recipient mice. 15 hrs later their LN (A) were stained with anti-B220-PE-Texas red (red) and anti-CD3-APC (blue) and spleens (B) were stained with anti-B220-APC (blue) (upper pictures) or anti-MOMA-1-biotyl/streptavidin-Alexa647 (blue) (lower pictures) and analyzed by confocal microscopy (see Methods). The graph in (A) represents the number of CFSE+ cells per section of LN. The graph in (B) represents the number of CFSE+ cells per splenic white pulp. Number of white pulps analyzed per mouse was > or  = 2. Data are from 8 (control), 3 (Mela-bearing) and 4 (Amela-bearing) mice. Statistics are as described in Methods.

To examine whether migration of naïve TL in TDLN is affected, CFSE-labeled naïve CD8 TL were i.v. injected in control and in Mela- and Amela-developing mice. Mice were sacrificed 15hrs later and LN sections were analyzed by immunohistology. Staining with anti-CD3 and anti-B220 monoclonal antibody (mAb) to detect, respectively, the T-zones and B-zones of the LN was performed (Fig.2A). In control and Mela-bearing mice, defined B- and T-zones were apparent and CFSE-labeled TL co-localized with the endogenous TL. In Amela-bearing mice, however, some B-zones were visible, but no defined T-zones were apparent. CFSE-labeled TL were distributed throughout the LN. Analysis of LN sections from different mice showed that the total number of TL per section area in LN of the hosts was significantly reduced in Amela-bearing mice (graph in Fig.2A).

The migration of the CFSE-labeled TL in the spleen was also evaluated by immunohistology (Fig.2B) on sections labeled with anti-B220 or MOMA-1 antibody identifying metallophilic macrophages adjacent to the marginal zone of the spleen, which is the region at the interface between non-lymphoid red pulp and lymphoid white-pulp [28]. In control and Mela-bearing mice transferred naïve TL localized predominantly to the white pulp area outlined by MOMA-1 positive cells. In contrast, in Amela-bearing mice transferred TL had a diffuse distribution throughout the spleen. Data for a number of splenic sections from different mice are shown (Fig.2B).

We next examined expression of CCR7, the main chemokine receptor necessary for appropriate location of TL to SLO and found similar levels on TL present in control LNs, Mela- or Amela-TDLN (Fig.S2).

The results indicate that localization of endogenous as well as of adoptively transferred naïve TL is perturbed in TDLN and in spleens of induced Amela-bearing mice. Thus, this defect did not appear to be intrinsic to the TLs, but might result from SLO disorganization.

### Disruption of lymphoid organization and decrease of T- and B-zones in mice developing Amela-melanomas

**Figure 3:**
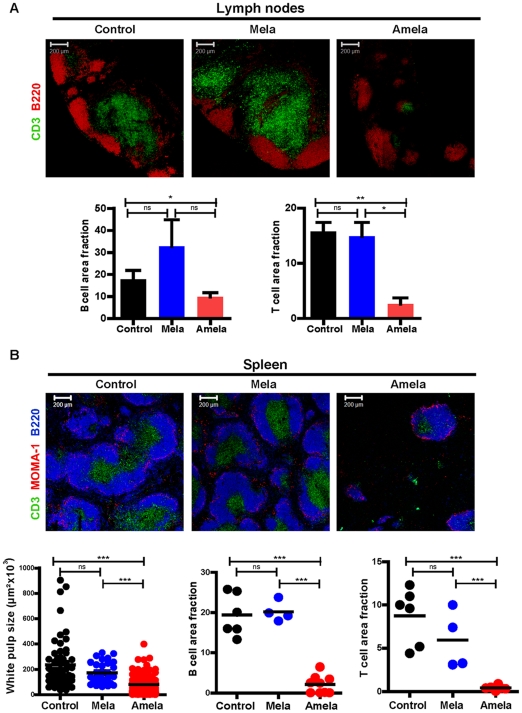
Lymphoid organization in developing Amela-melanomas is disrupted. Analysis of LN (A) and spleen (B) from control, Mela- and Amela-bearing mice. (A) In LN, B- and T-zones were identified with anti-B220-PE-Texas red (red) and anti-CD3-FITC (green), respectively. Graphs represent the density of B (left) and T (right) cells, determined by pixel quantification. For tumor-bearing mice, TDLN are shown. Data are from 7 (control), 4 (Mela-bearing) and 4 (Amela-bearing) mice. (B) Splenic B-zones, T-zones and marginal zones were identified with anti-B220-APC (blue), anti-CD3-FITC (green), and anti-MOMA-1/goat anti-rat-Alexa647 (red), respectively. The white pulp areas are delineated by MOMA-1 expression. One graph (left) shows the size of white pulp areas in the different mice (each symbol represents a white pulp area). Density of B (middle graph) and T (right graph) cells were determined by pixel quantification. Data are from 6 (control), 4 (Mela-bearing) and 9 (Amela-bearing) mice.

To further evaluate the defect in organization of SLO in Amela-bearing mice, we assessed quantitatively T and B cell areas in a number of sections of TDLN from different tumor-bearing mice or of LN from different control mice using ImagJ software. While T cell areas represent more than 15% of LN area in control or in Mela-TDLN, they represent less than 5% of the area in Amela-TDLN (graph in Fig.3A). No statistically significant decrease in B cell area fractions was observed in Amela-TDLN (graph in Fig.3A).

For spleen sections (Fig.3B), quantification of white pulp size (graph in Fig.3B) showed a significant reduction in Amela-bearing mice compared with control or Mela-bearing mice correlated with a decreased area fraction of T and B cells (graphs in Fig.3B). However, the total number of splenic B and T cells was not decreased in Amela-bearing mice (Table S1). This observation means that in Amela-bearing mice splenic TL did not form follicles, as in control mice, but were dispersed throughout the spleen, and in particular in the red pulp.

### Loss of gp38+ FRC-associated CCL21 in spleens and TDLN of mice developing Amela-melanomas

**Figure 4:**
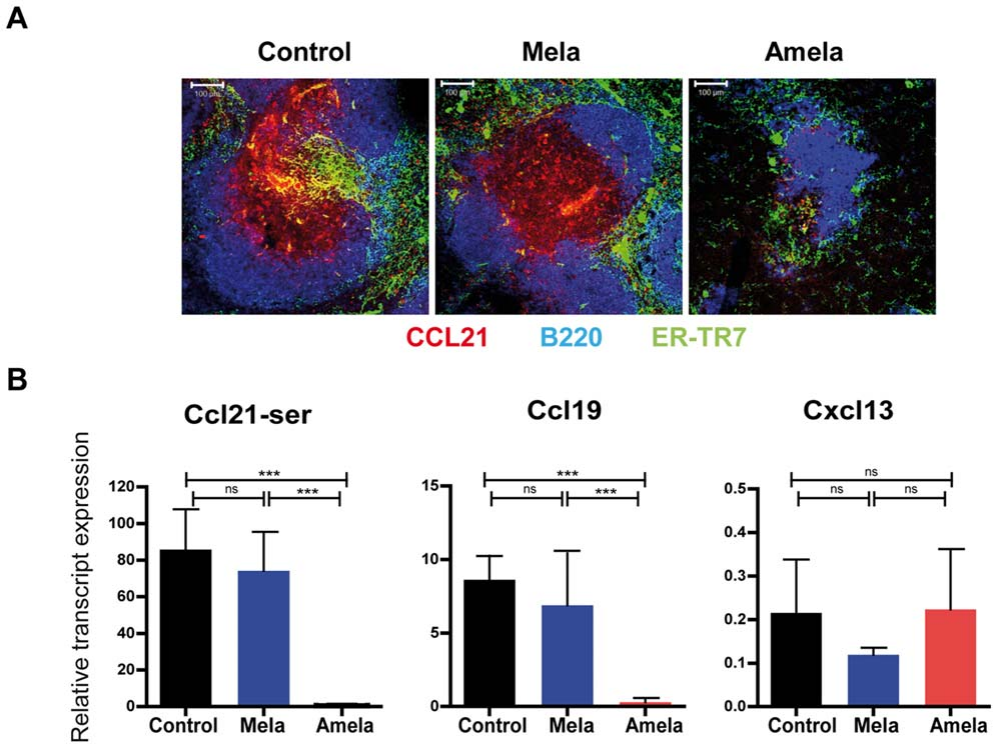
Decrease in splenic lymphoid chemokines in mice developing Amela-melanomas. Spleen sections from control, Mela- and Amela-bearing mice stained in (A) with anti-B220-APC (blue) to detect the B-zone, ER-TR7/chicken anti-rat-Alexa488 (green) to detect the FRC network and anti-CCL21/donkey anti-goat-Alexa546 (red). (B) Expression of *ccl21a* encoding Ccl21-Ser, *ccl19* and *cxcl13* relative to *tbp* transcripts measured by QRT-PCR in total spleens of control, Mela- or Amela- bearing mice. Means (+/− s.e.m.) for independent samples (N = 10; 8; 14 for *ccl21a*; N = 5; 4; 8 for *ccl19*; N = 3; 3; 9 for *cxcl13*) from spleens of control, Mela- or Amela- bearing mice, respectively, A is representative of 5 mice per group.

B- and T-zones of SLO are organized by the expression of homeostatic chemokines by defined fixed stromal cells: FDC secreting CXCL13 and FRC secreting CCL19 and CCL21, respectively in B- and T-zones [5], [29]. FRC, as many fibroblasts, express a molecule recognized by mAb ER-TR7 [30], as well as podoplanin (gp38) [31]. We analyzed expression of CCL21 on sections of spleens together with ER-TR7 and B220 staining (Fig.4A). Interestingly we observed an important decrease in expression of CCL21 in spleens from Amela-bearing mice. QRT-PCR analysis (Fig.4B) further showed reduced expression of both CCL21-Ser and CCL19 transcripts in spleen samples from Amela-bearing mice, whereas expression of CXCL13 transcripts was not affected.

**Figure 5:**
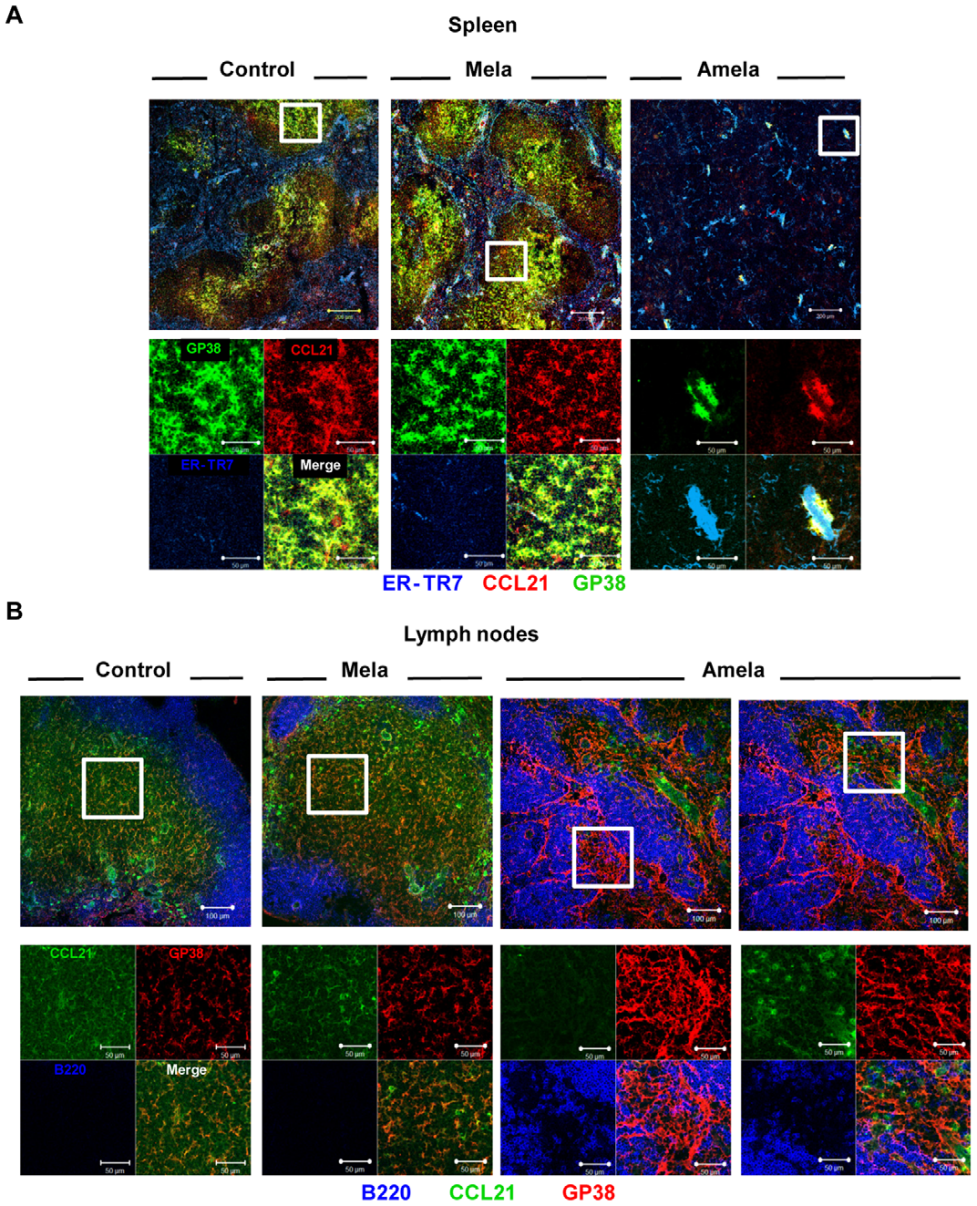
Loss of gp38+ FRC-associated CCL21 in spleens and TDLN of mice developing Amela-melanomas. In (A) spleen sections were stained with ER-TR7/chicken anti-rat-Alexa647 (blue), anti-gp38/donkey anti-goat-Alexa488 (green) and anti-CCL21-biotyl/SA-Alexa546 (red). In (B) LN sections were stained with anti-B220-Alexa647 (blue), anti-gp38/donkey anti-goat-Alexa546 (red) and anti-CCL21-biotyl/SA-Alexa488 (green). A and B are representative of 3 mice per group. Lower images show magnifications of the fields highlighted in the upper images and show stainings with each of the antibodies separately and merged, as indicated.

To address the question whether the decrease in CCL21 is the consequence of a reduction in T-zone associated FRCs, we performed immunohistology for detection of gp38 and CCL21 together with ER-TR7 on splenic sections (Fig.5A). Results showed that the density of gp38+ cells was greatly decreased, as was CCL21 detection in spleens of Amela-bearing mice as compared to those of control or Mela-bearing mice. A similar analysis performed on LN sections showed co-localization of CCL21 staining with gp38 expression in the T-zones for control LN and Mela-TDLN (Fig.5B). In Amela-TDLN, however, the density of gp38+ cells was increased, but they were not associated with CCL21 expression. These strongly gp38+ cells probably corresponded to endothelial cells and were not restricted to the T cell zone, as reported [6]. Punctate CCL21 staining was detected in areas that are distinct from the gp38-associated CCL21 staining observed in control LN and in Mela-TDLN (Fig.5B).

Altogether, our results show that the reduced T-zones in spleen and TDLN, are associated with a reduction in CCL21+gp38+ FRCs.

### The architecture of SLO is disrupted in mice developing Amela-melanomas

**Figure 6:**
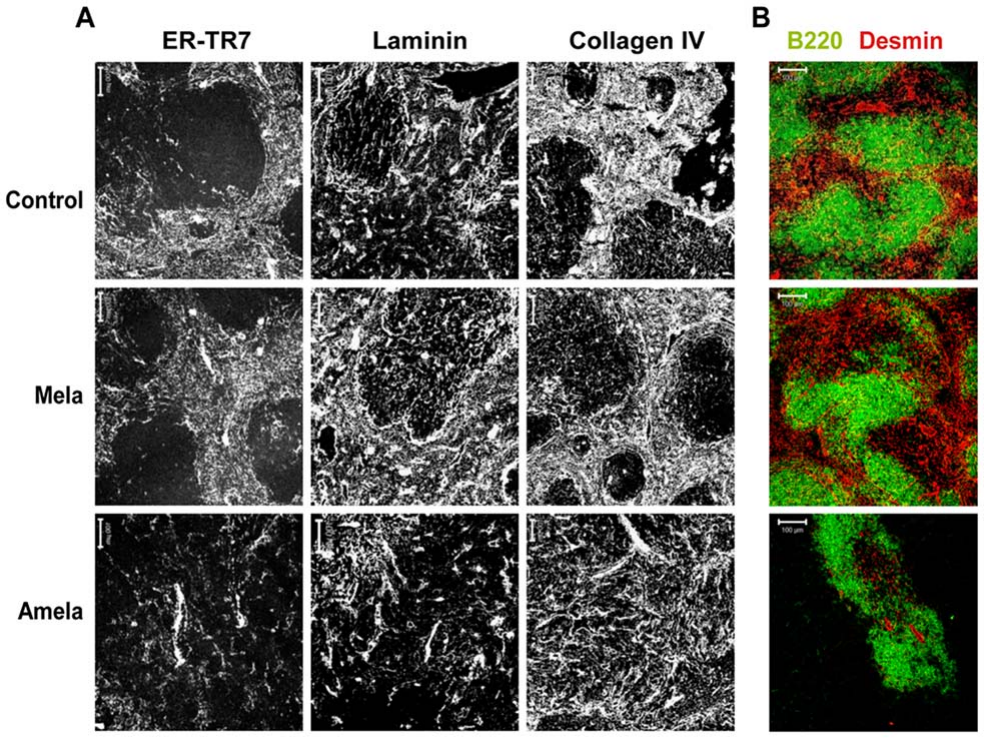
Splenic architecture is disrupted, with loss of contractile fibroblasts, in mice developing Amela-melanomas. Spleens from control, Mela-, and Amela-bearing mice stained in (A) with ER-TR7/chicken anti-ratAlexa488 (left), anti-laminin/donkey anti-rabbit-Alexa488 (middle), and anti-collagen IV/donkey anti-rabbit-Alexa488 (rigth) to detect stromal networks, and in (B) with anti-B220-FITC (green), anti-Desmin/donkey anti-rabbit-Alexa555 (red). Representative of 5 mice per group.

FRC are also implicated in production of reticular fibers that organize SLO [32]. We next addressed the question of the integrity of the SLO architecture in Amela-bearing mice. FRC surround collagen fibers and form conduits in which low molecular weight molecules from the periphery are trapped [33], [34]. The surface of this conduit also guides TL migration [7]. Thus, it is important for the adequate localization of cells in SLO that this complex network be well conserved. By staining sections of SLO for different elements of the FRC network (Fig.6A), we observed in spleens of both control and Mela-bearing mice that the ER-TR7 positive network defined a dense group of fibers localized in the red pulp and a less dense network in the white pulp, as described (reviewed in [29]). However, in Amela-bearing mice this network seemed to be less dense, as well as discontinuous, with shorter fibers. We analyzed expression of other molecules that compose the network, such as laminin and collagen IV. The results were similar to those seen for ER-TR7, in particular for laminin. The different staining experiments show that the splenic stromal cell network is disrupted in Amela-bearing mice (Fig.6A).

FRC also have a contractile function and share certain features with smooth muscle cells or myofibroblasts [6], [29]. Consequently, we investigated the possibility that alteration of the stromal cell network might be associated with reduction of cells with contractile activity. Staining for desmin expression (Fig.6B), indeed, revealed a loss of desmin^+^ cells in spleens of Amela-bearing mice. Thus, in association with the decrease in the area of gp38^+^ cells, which in spleens characterize FRC of T-zones, we observed a loss of FRC, in particular, the desmin+ fraction of FRC. In addition to being enlarged, the splenic red pulp of Amela-bearing mice is thus also altered phenotypically. To rule out the possibility that metastasis of melanoma, although not visible, might induce the disruption of the splenic architecture, we tested for presence of transcripts of the oncogene H-Ras^G12V^ which is expressed by the induced melanomas (QRT-PCR, data not shown). However, we failed to detect expression of H-Ras^G12V^ transcripts in spleens from Amela-bearing mice, so this hypothesis seems unlikely.

The LN stromal network, although reminiscent of that of the spleen, presents some differences. As for the splenic red pulp, the LN medulla presents a dense ER-TR7+ network whereas the LN T-zone ER-TR7+ network is less dense [5], [29]. In LN, however, desmin is highly expressed in the T-zones ([6], Fig.S3A–B). In Amela-bearing mice, the discontinued network fibers observed in the spleen are not consistently observed in the TDLN. However, in these mice we always noticed an enlargement in the medullar and a decrease in the T-zone networks in TDLN. Furthermore the desmin+ER-TR7+ fibers characteristic of T-zones in control and Mela-TDLN are reduced and morphologically different in Amela-TDLN (Fig.S3B).

### CD11b^+^Gr1^+^ iMC localized in LN medulla and spleen red pulp interact with the stromal network in mice developing Amela-melanomas

**Figure 7:**
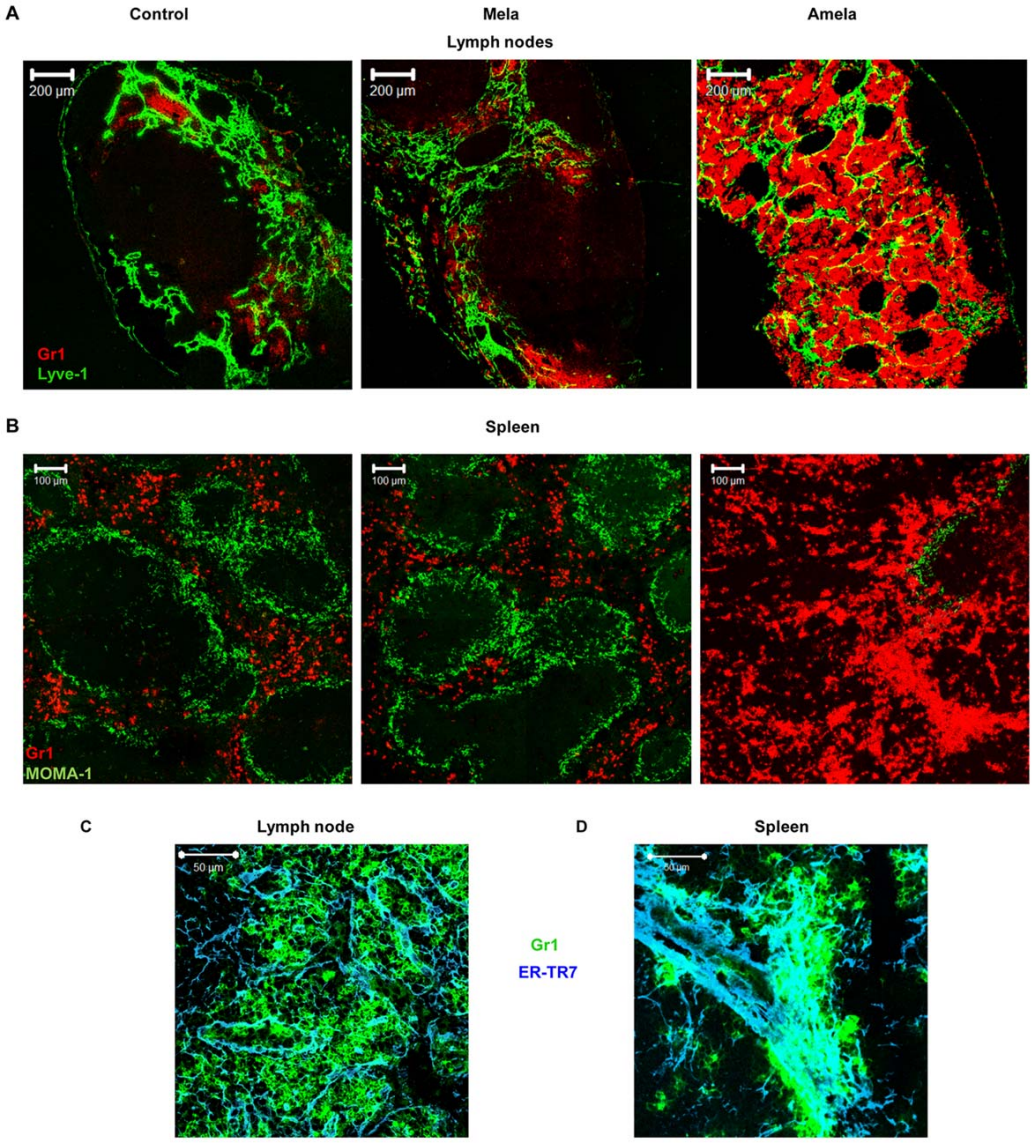
Gr1^**+**^CD11b^**+**^ cells recruited in SLO interact with the stroma in mice developing Amela-melanomas. LN (A) or spleen (B) sections from control, Mela- and Amela-bearing mice were stained with anti-Gr1-PE (red) and anti-Lyve-1-biotyl/streptavidin-FITC (green) to detect LN medullas or for MOMA-1 (green) to delineate splenic white pulp areas, respectively. LN (C) or spleen (D) sections from Amela-bearing mice were stained with anti-Gr1-APC (green) and anti-ER-TR7/chicken anti-rat-Alexa647 (blue). Magnification showing a cluster of Gr1^+^ cells on the stromal network is shown.

We reported that CD11b^+^Gr1^+^ cells accumulate in SLO of Amela-bearing mice [17]. To evaluate the potential impact of these iMC on SLO organization in Amela-bearing mice, we analyzed localization of CD11b^+^Gr1^+^ cells in SLO and, in particular, investigated whether accumulation of these cells correlated with alteration of SLO architecture (Fig.7A–D).

Sections of LN and spleens were stained with antibodies to Gr1. For LN we used the Lyve-1 marker of lymphatic endothelial cells to define the medulla, and for spleens the MOMA-1 marker to localize the white pulp (Fig.7A–B). Most Gr1^+^ cells were localized in LN medulla and in splenic red pulp in all samples. As we observed a destruction of the stromal cell network in SLO of Amela-bearing mice, we wondered whether CD11b^+^Gr1^+^ cells could interact with the network. To this end we stained LN and spleen sections for ER-TR7 and Gr1 markers (Fig.7C–D). We observed that Gr1^+^ cells were associated with remnants of the stromal cell network, in particular in the spleen (Fig. 7D), as described for lymphocytes that use it for their guidance and motility [7], [8].

CD11b^+^Gr1^+^ cells also appeared to form clusters in spleens and Amela-TDLN (Fig.S4), possibly the consequence of either their proliferation around the stromal network or of their recruitment. To discriminate between these two possibilities, we stained spleen, LN and control skin or Amela-tumors for the proliferation marker Ki-67. No colocalization between the Ki-67 and Gr1+ markers was detected in spleen, LN or tumor/tissue (Fig.S4). This observation suggests that the clustering of Gr1+ cells in SLO is due to the recruitment of iMC cells from the bone marrow in Amela-bearing mice. In the spleen, they are localized in the red pulp where they closely interact with the stromal cell network. In the Amela-TDLN, the CD11b^+^Gr1^+^ cells are found in the medulla, where they may have migrated either from the blood or from the tumor tissue. This observation is reminiscent of that described for neutrophils [35] in a model of parasite infection. The fact that principally TDLN are affected in their organization and in recruitment of iMC may suggest that LN are less influenced by the systemic effects of TDF, but are rather under the local influence of TDF and recruited cells.

## Discussion

Defects in DC differentiation have been observed in both cancer patients and tumor bearing mice [36]. Evidence exits for a systemic influence of TDF on myeloid cell differentiation [13], [19]; other explanations for the paucity of mature DC in TDLN include DC killing by Treg [37] and down-regulation of CCR7 surface expression, required for DC migration from skin to LN [26]. However, these studies failed to analyze distinct DC subpopulations differing in their origin and level of CCR7 expression [23].

Analysis of these subpopulations in TDLN reported here in mice with autochthonous inflammatory melanomas revealed selective reduction in populations of MigDC compared to lymphoid tissue-ResDC. The reduced fraction of MigDC migrating to TDLN 20 hrs after skin painting is consistent with a defect in their migration and/or homing to LN rather than (or in addition to) a systemic perturbation of DC differentiation in these mice. No evidence for CCR7 down-modulation was observed in any MigDC subpopulation in the present study.

Migration of skin-derived DC from afferent lymphatics into LN T-zones is guided by CCL19 and CCL21-ser (encoded by gene *ccl21a*) expressed by T-zone FRC [6]. In plt/plt (plt, paucity of LN T cells) mice, which lack expression of CCL19 and CCL21-ser, MigDC are strongly reduced in skin-DLN despite expression of CCL21-leu, encoded by gene *ccl21c* by peripheral lymphatic vessels [27], [38]. In the autochthonous Amela-melanoma model, splenic reduction in CCL19 and CCL21 transcripts was associated with loss of gp38+ FRC, generating a phenotype similar to that of plt/plt mice with a defect in recruitment of naïve TL. In TDLN gp38+ cells were abundantly detected, which probably corresponded to lymphoid endothelial cells [6]. These cells were not associated with CCL21, as is the case for the gp38+ FRCs found in T-zones of control LN and these TDLN presented defects in recruitment of both CCR7**^+^** MigDC and TL.

A progressive decrease in CCL21 production in TDLN of mice with growing transplanted melanomas was also previously associated with impaired recruitment of naïve TL in LN, independent of LN metastasis [39]. Suppression of CCL21 production was also reported in TDLN of patients with metastatic melanoma [40]. Disruption of LN architecture as a result of down-regulation of both CCL21 and CXCL13 appeared to increase the virulence of Salmonella [41].

In the Amela-melanoma model, reduction of T-zones was associated with a loss of gp38+ FRCs and CCL21 in those zones. In addition, marked invasion of SLO with iMC was observed. In a previous study [39], Gr1^+^ polymorphonuclear cells appeared to invade the medulla, upper cortex and capsule of transplanted melanoma-TDLN with decreased CCL21 expression.

Our study reveals that important alterations of SLO architecture occur during autochthonous inflammatory tumor development. It identifies multiple defects apparently induced by Amela-tumors that could explain the reduction in T-zone area in SLO of tumor-bearing mice. Of note, adaptive immune responses are generally associated with a down-regulation of CCL21 production by FRC [42], but this effect was transient and was attributed to IFN-γ, a cytokine that is not produced in Amela-bearing mice [17].

Other mechanisms may be involved in the context of inflammatory tumor development. The Amela tumor secretes several factors implicated in SLO remodeling, including VEGF and FGF-2 [17]. These TDF could contribute to the important lymphangiogenesis in Amela-TDLN, since stromal cells present in the LN medulla and splenic red pulp of Amela-bearing mice might express VEGFR2 (Fig.S5). Systemic effects of VEGF on splenic organization were reported in mice transplanted with tumors over-expressing VEGF [43]. The increase in size of the splenic red pulp or LN medulla could contribute to decreases of splenic white pulp or LN T-zones, respectively. Additionally, a paucity of TL in TDLN has recently been correlated with iMC/MDSC-induced shedding of CD62L, the L-selectin that directs naïve TL into LN [44]. This activity was linked to increased expression of the protease ADAM17 by the iMC/MDSC [44]. Down-regulation of CD62L from TL also occurred in mice with inflammatory melanoma (Fig.S6A), We even observed shedding of CD62L from naïve polyclonal TL 24hrs after their transfer in Amela-bearing mice (Fig.S6B). Since L-selectin is not implicated in TL homing to the spleen, additional mechanisms are probably at play. In particular, other protease activities have recently been found to be exerted by iMC/MDSC [45]. We observed that transcripts for MMP9, cathepsin G and neutrophil elastase are over-expressed in spleens of Amela-bearing mice (Fig.S6C). Whether the corresponding activities may contribute to the destruction of SLO stromal cell networks remains to be established. Neutrophils, which are known to degrade tissues by releasing matrix metalloproteinases, have been described to participate in LN remodeling during parasite infection [35]. We may speculate that iMC/MDSC might similarly contribute to SLO remodeling in the case of inflammatory tumors. Other mechanisms such as iMC-induced apoptosis may also contribute to reduction of FRC, notably by limiting factors involved in FRC homeostasis.

A striking result from this study is that inflammatory tumors perturbed SLO architecture and rendered SLO non-receptive for DC and TL, while iMC clearly entered into these perturbed SLO. It has been difficult to directly address the question of the role of iMC in destruction of SLO architecture by their adoptive transfer into hosts devoid of tumor influence, since these cells migrated to the bone marrow, but not to the spleen and LN in these hosts (Fig.S7A). Similarly, treatment with anti-Gr1 mAb failed to eliminate the Gr1+CD11b+ population from aggressive inflammatory melanoma-bearing mice (Fig.S7B). This failure may be related to the production by the Amela-tumors of factors such as G-CSF implicated in recruitment of myeloid precursors from the bone marrow, and of chemokines such as CCL2 known to recruit iMC [17]. On the other hand, injection of isolated chemokines (CCL2) / growth factors (VEGF) failed to reproduce the disrupted phenotype of SLO observed in inflammatory melanoma-bearing mice, although they might be contributing factors [43], [46].

The recognition that SLO of inflammatory tumor-bearing hosts are poorly receptive to CCR7-dependent migration of MigDC and naïve TL may be relevant for proposed vaccination or adoptive TL therapies applied to such tumors. The importance of selecting tumor-specific TL with capacity for long-term survival for adoptive therapy has recently been stressed [47]. Our data indicate the additional importance of selecting TL with appropriate tissue/tumor migratory properties, rather than CCR7^+^ naïve or memory TL that migrate to the T-zones of SLO, for treatment of the autochthonous inflammatory tumors that we study. Indeed, these tumors, in contrast to transplanted tumors [48], are poorly infiltrated by either naïve or weakly activated transferred TL [17], [49]. Our studies are now aimed at designing anti-tumor TL effectors with characteristics allowing them to migrate directly into tumors and to resist the immunosuppressive tumor microenvironment. This treatment, in combination with therapeutic antibodies blocking TDF which either act directly on SLO (such as VEGF, FGF-2), or act indirectly via the mobilization of iMC/MDSC [15], may further contribute to restore the efficacy of the host's endogenous immune system.

## Materials and Methods

### Ethics statement

All procedures were approved by the Regional “Provence-Alpes-Cote d'Azur” Committee on Ethics for Animal Experimentation (authorization: #13.21, date: 11/02/2000) and were in accordance with French and European directives.

### Mice

We used TiRP-10B;Ink4a/Arf^flox/flox^ mice [16] backcrossed to strain B10.D2/nOlaHsd (Harlan, Gannat, France) [17] that develop either Mela- or Amela-melanomas after 4OH-tamoxifen (Sigma) treatment ([17] and Text S1). Examples of mice with induced Mela- or Amela-melanomas used in this study are shown in Fig.S8. Age-matched TiRP-10B negative Ink4a/Arf^flox/flox^ control mice, treated with 4OH-tamoxifen as the experimental mice, never developed tumors because of the absence of the TiRP-10B transgene [17]. Mice were housed under specific pathogen-free conditions.

### Immunohistology

Spleens, LN, skin or tumors were snap-frozen in Tissue Tek (Sakura Finetek). Serial frozen sections (5- to 10-µm) were fixed in acetone and stained with the indicated antibodies. Confocal microscopy and image processing were performed, respectively, with a Zeiss LSM 510 META microscope and Zeiss LSM software. Immunofluorescence was quantified using NIH ImageJ software for the determination of relative densities of expression of given markers within fixed section areas (fraction area).

### Adoptive transfer experiments

Naïve TL isolated from LN and spleens of B10.D2 mice were labeled with the intra-cytoplasmic dye, carboxyfluorescein succinimidyl ester (CFSE) (Molecular Probes) and 5×10^6^ of these cells in 100 µl PBS were injected i.v. in mice, which were sacrificed 15 hrs later for analysis of migration by immunohistology.

### DC isolation

LNs were cut in small pieces and incubated for 20 min with a mixture of collagenase II (Worthington Biochemical) and DNAse (Sigma-Aldrich) as described [20].

### Assay of skin-derived DC migration

TRITC (Tetramethylrhodamine-5-(and-6)-isocyanate, Molecular Probe (Eugene, OR)), dissolved in a 50∶50 (v/v) acetone-dibutylphtalate mixture, was applied (30 µl of 10% TRITC) on the tumor and shaved skin around the tumor for Amela-bearing mice and on the equivalent shaved skin area of control mice. Shaving was performed at least 24 hrs before skin-painting. Mice were killed 20 hrs after painting and DC were isolated from LNs and analyzed by fluorescence-activated cell sorter (FACS).

### Flow cytometry

Before staining, cells were preincubated on ice with 2.4G2 mAb to block Fc-receptors. For analysis of DC subpopulations, LN cell suspensions were gated on CD11c^+^NK1.1^−^CD25^−^CD45R^−^ cells and autofluorescent cells were gated out using the AmCyan channel. FACS analysis was performed using the LSRII and data were analyzed using DIVA software.

### Antibodies

For FACS analysis, mAbs specific for CD161c (NK1.1)-APC-H7, CD45R (RA3-6B2)-APC-H7, CD25 (PC61)-APC-H7, CD197/CCR7 (4B12)-Biotin were from BD (Biosciences Pharmingen) and those specific for CD11c (N418)-PECy5.5 and MHC class II (M5/114)-A700 were from eBioscience. For CD207/langerin intracellular staining, mAb (929F3)-A647 (Abcys) was used with the Cytofix/Cytoperm kit from BD.

For immunohistology, ER-TR7 mAb specific for an unknown FRC-secreted molecule was from BMA. MAbs to Gr1 (RB6-8C5), CD11b (M1/70), CD3 (2C11) and B220 (RA3-6B2) were from BD, mAbs to Desmin, Collagen IV and Laminin from Abcam. Anti-MOMA-1 (Cerdalane laboratories), anti-CCL21, anti-gp38 and anti-Lyve-1 (R&D system) antibodies were used. For uncoupled primary antibodies, incubations were at 4°C overnight, followed by secondary antibody for 45 min at room temperature. For directly coupled antibodies, incubations were for 45 min at room temperature. All combinations of primary and secondary antibodies were pre-tested for specificity of labeling.

### Quantitative RT-PCR

Total RNA was isolated from whole spleens (conserved at −80°C in RNA later) using RNeasy Mini Kit column purification and digestion with RNase free DNaseI according to the manufacturer's protocols (Qiagen). cDNA was generated using the SuperScript® first-strand synthesis system for RT-PCR according to the manufacturer's instruction (Invitrogen). Quantitative real time RT-PCR was performed with an Applied Biosystem Prism 7500 fast real time PCR system using SYBR green PCR Master Mix (Applied Biosystems). Results were normalized on the housekeeping gene *tbp* (TATA binding protein). Oligonucleotide sequences are reported in Table S2.

### Statistics

Statistical analyses were performed with the Student's *t* test using GraphPad and two-tailed *P* values are given as: (*) *P*<0.1; (**) *P*<0.01; and (***) *P*<0.001; (ns) *P*>0.1.

## Supporting Information

Figure S1 **Comparaison of representation of DC subsets in LN from control, Mela- and Amela-bearing mice**. Analysis of DC subpopulations in TDLN (left) or non-draining LN (NDLN) (right) from Amela- (A) or Mela- (B) bearing or LN from control (C) mice. After gating on CD11c^+^NK1.1^−^CD25^−^CD45R^−^ cells, CD11c versus MHCII staining identifies MigDC (CD11c^+^MHCII^high^) and ResDC (CD11c^high^MHCII^intermediate^) and shows a relative depletion of the MigDC population selectively in TDLN of Amela-bearing mice. (D) Immunohistology of sections from control LN, Mela-TDLN and Amela-TDLN showing anti-MHCII (white), anti-B220 (green; B cells) and anti-CD3 (blue; T cells).
(PDF)

Figure S2 **Similar level of CCR7 expression on TL from control, Mela- or Amela-bearing mice.** Spleen cells harvested from control, Mela- or Amela-bearing mice were stained with anti-CD3 and anti-CCR7 mAb. FACS analysis of CCR7 expression within the CD3+ fraction is shown.
(PDF)

Figure S3 **Modifications of the stromal network in Amela-TDLN.** LN sections from control mice and TDLN from Mela- and Amela-bearing mice were stained with (A) anti-B220-FITC (green), anti-Desmin/donkey anti-rabbit-Alexa555 (red) or (B) with anti-ER-TR7/chicken anti-rat-Alexa647 (green) and anti-Desmin/donkey anti-rabbit-Alexa555 (red). In (B) mice had received 10^6^ CFSE-labeled B10.D2 TL (withe), as in Fig.2, 20hrs before their sacrifice. In the magnification (left), the TL (withe) can be seen to interact with the Desmin+ER-TR7+ FRC in the Mela-TDLN.
(PDF)

Figure S4 **Gr1^+^CD11b^+^ iMCs are recruited to SLOs and tumor in Amela-bearing mice.** Analysis of spleen (left), LN (middle) and skin or tumor (right) sections from control mice (upper) and Amela-bearing mice (lower). The sections were stained for the proliferation marker Ki-67 (green), Gr1 (red) and the nuclei marker Topro3 (blue) as described (Soudja et al. 2010).
(PDF)

Figure S5 **Stromal cells present in the splenic red pulp of Amela-bearing mice express the VEGFR2.** Spleen sections from control and from Amela-bearing mice were stained with anti-collagen IV antibody (red) and with goat anti-mouseVEGFR2 (Flk-1) antibody from R&D Systems (green). Single stainings and merge images are shown. A magnification of the merged image is shown (far rigth).
(PDF)

Figure S6 **Potential mechanisms implicated in the disruption of SLO in Amela inflammatory tumor-bearing mice.** (A) FACS analysis of CD62L expression on CD8 TL from LN and spleens of control mice (black), Mela- (blue), or Amela- (red) bearing mice. Mean fluorescence intensity values were normalized to those of the control samples. (B) TL from CD45.1 B10.D2 mice were purified and transferred in control mice, Mela- or Amela-bearing mice (all of which are CD45.2). 24 hrs later mice were sacrificed. FACS analysis of CD62L expression on transferred TL from control mice (black line), Mela- (blue line) or Amela- (red line) bearing mice is shown for LN (left) and spleen (right) samples. Gray lines are for unstained samples. (C) Quantitative RT-PCR for MMP9 (*Mmp9*), Cathepsin G (*Ctsg*) and Elastase-2 (ELA-2, *Cela2a*) transcript expression normalized to TBP (*Tbp*) mRNA expression in whole spleens from control, Mela- or Amela-bearing mice.
(PDF)

Figure S7 **iMCs retrieved from spleens of Amela-bearing mice migrate to the bone marrow (BM) in tumor-free mice and anti-Gr1 mAb treatment fails to deplete them from Amela-bearing hosts.** **(A)** Spleen cells from Amela-bearing mice rich in iMCs were labeled with CFSE 10 µM and transferred in tumor-free or in induced Amela-bearing mice. 20 hrs later LN, spleen and BM were harvested and FACS analysis was performed on CFSE+ gated cells. % of CFSE+ cells expressing Gr1 and CD11b (Gr1CD11b) or CD3 (T cells) is indicated as the mean +/− s.d. for 3 mice per group. Enrichment in Gr1+CD11b+ cells in spleens and in LN was observed only in Amela-tumor bearing mice. (B) Control and induced Amela-bearing mice were treated with 300 µg anti-Gr1 mAb (RB6-8C5) injected i.p. every 2 days for 6 days, were sacrificed at day 8 and LN, spleen and BM cells were analyzed by FACS for expression of CD11b and Ly-6G mAb (1A8), which recognizes an epitope of the Ly-6G/Gr1 molecule that is distinct from that recognized by mAb RB6-8C5. High percentages of CD11b+Ly6−G+ (iMCs) were still present in LN and spleen of Amela-bearing mice. Representative of two mice per group.
(PDF)

Figure S8 **Examples of TiRP-10B Ink4a/Arf^flox/flox^ B10.D2 mice with induced pigmented Mela- or amelanotic Amela-melanomas used in this study.**
(PDF)

Table S1 **conserved numbers of TL in spleens of mice developing Amela-melanomas.** (a) Spleen cells harvested from control, Mela- or Amela-bearing mice were counted and stained for CD45, CD4, CD8 and B220 expression by FACS as described (Soudja et al. 2010). N =  number of mice analyzed. (b) % CD45+ cells among live spleen cells are given +/− SD. Corresponding cell numbers are given in bold. (c) % CD8 TL among CD45+ spleen cells +/− SD. Corresponding cell numbers are given in bold. (d) % CD4 TL among CD45+ spleen cells +/− SD. Corresponding cell numbers are given in bold. (e) % B220^+^ cells among CD45+ spleen cells +/− SD. Corresponding cell numbers are given in bold.
(DOC)

Table S2 **Murine primer sequences used in the real-time QRT-PCR amplifications shown in** **Fig.4B** **and in Fig.S6C.**
(DOC)

Text S1 **Mice.** On the B10.D2 (B10.D2/nOlaHsd, H-2^d^) background, the TiRP-10B Ink4a/Arf^flox/flox^ B10.D2 mice housed in the CIML facility develop, after 4OH-tamoxifen treatment (injection s.c. twice two weeks apart of 4 mg 4OH-tamoxifen dissolved in ethanol and brought to 20 mg/ml with autoclaved sunflower oil as described (Huijbers et al. 2006)), single melanomas, either strongly pigmented (Mela) or unpigmented (Amela) with similar latency (on average 160 days) (Soudja et al. 2010). The incidence of Amela development was about 4 fold higher than that of Mela tumors. Mela- and Amela-tumor bearing mice analyzed in the present study each carried a single tumor, the size of the latter being on average twice that of the former. For examples, see Fig.S8.
(DOCX)
