# Supplementary figures and images for: Disrupted Lymph Node and Splenic Stroma in Mice with Induced Inflammatory Melanomas Is Associated with Impaired Recruitment of T and Dendritic Cells
Source: PLoS One. 2011 Jul 21;6(7):e22639. doi: 10.1371/journal.pone.0022639 (PMC3141075; doi:10.1371/journal.pone.0022639)

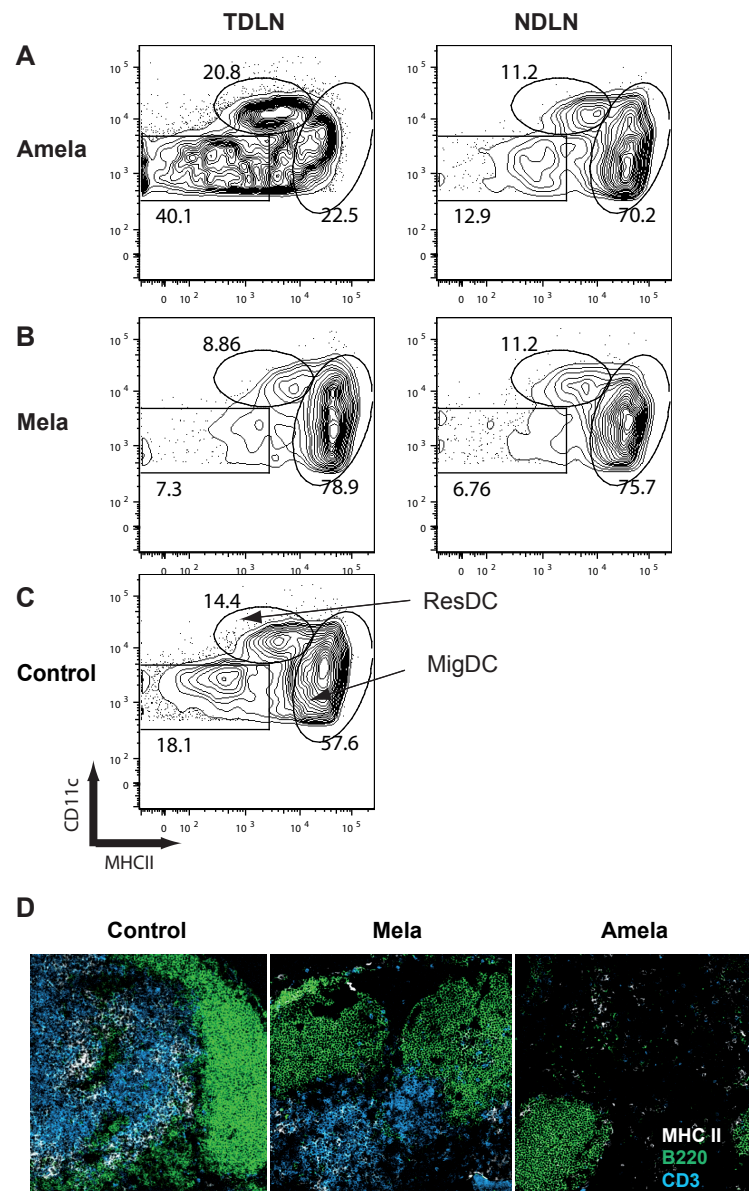

Supplemental figure 1

Supplement: Figure S1 — Comparaison of representation of DC subsets in LN from control, Mela- and Amela-bearing mice. Analysis of DC subpopulations in TDLN (left) or non-draining LN (NDLN) (right) from Amela- (A) or Mela- (B) bearing or LN from control (C) mice. After gating on CD11c+NK1.1−CD25−CD45R− cells, CD11c versus MHCII staining identifies MigDC (CD11c+MHCIIhigh) and ResDC (CD11chighMHCIIintermediate) and shows a relative depletion of the MigDC population selectively in TDLN of Amela-bearing mice. (D) Immunohistology of sections from control LN, Mela-TDLN and Amela-TDLN showing anti-MHCII (white), anti-B220 (green; B cells) and anti-CD3 (blue; T cells). (PDF) [file pone.0022639.s001.pdf]

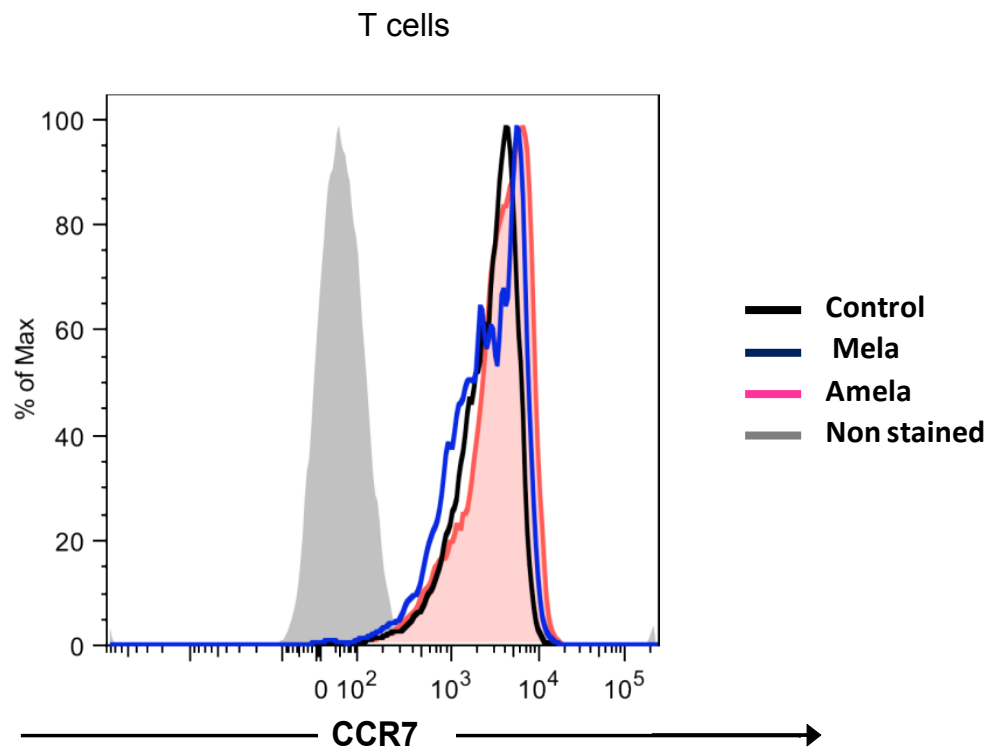

Supplemental figure 2

Supplement: Figure S2 — Similar level of CCR7 expression on TL from control, Mela- or Amela-bearing mice. Spleen cells harvested from control, Mela- or Amela-bearing mice were stained with anti-CD3 and anti-CCR7 mAb. FACS analysis of CCR7 expression within the CD3+ fraction is shown. (PDF) [file pone.0022639.s002.pdf]

**A**

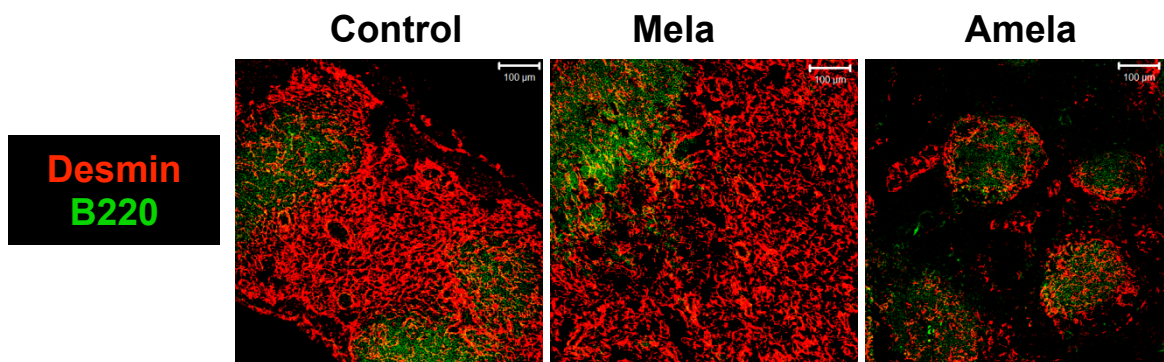

**B**

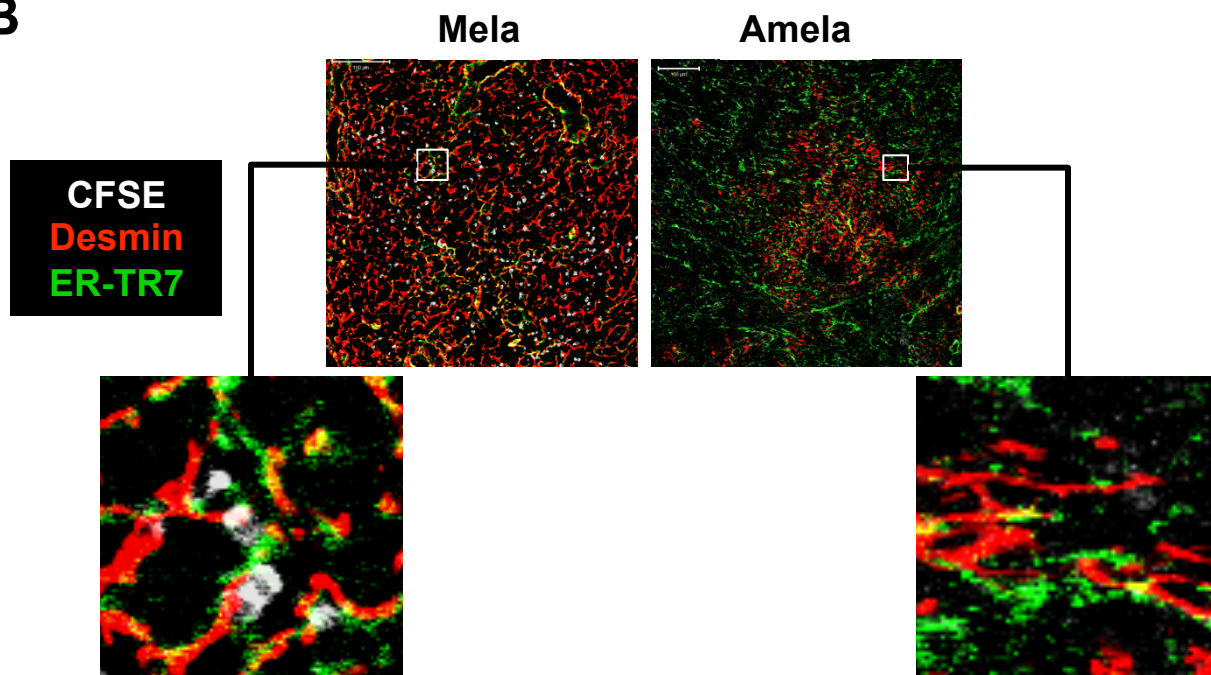

Supplemental figure 3

Supplement: Figure S3 — Modifications of the stromal network in Amela-TDLN. LN sections from control mice and TDLN from Mela- and Amela-bearing mice were stained with (A) anti-B220-FITC (green), anti-Desmin/donkey anti-rabbit-Alexa555 (red) or (B) with anti-ER-TR7/chicken anti-rat-Alexa647 (green) and anti-Desmin/donkey anti-rabbit-Alexa555 (red). In (B) mice had received 106 CFSE-labeled B10.D2 TL (withe), as in Fig.2, 20hrs before their sacrifice. In the magnification (left), the TL (withe) can be seen to interact with the Desmin+ER-TR7+ FRC in the Mela-TDLN. (PDF) [file pone.0022639.s003.pdf]

**Tropo3** **Ki-67** **Gr1**

**Control**

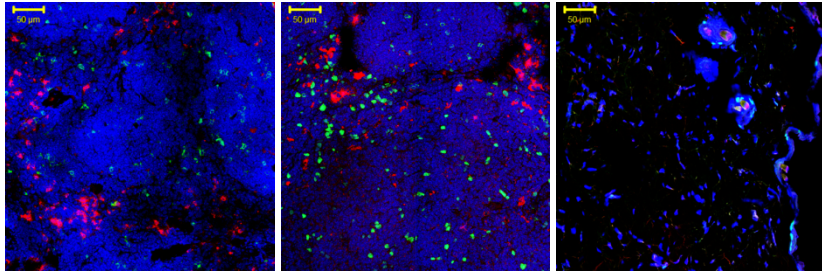

**Amela**

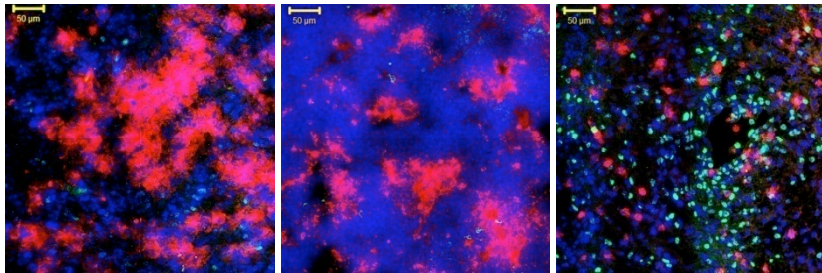

**Spleen**

**Lymph node**

**Tissue / tumor**

Supplemental figure 4.

Supplement: Figure S4 — Gr1+CD11b+ iMCs are recruited to SLOs and tumor in Amela-bearing mice. Analysis of spleen (left), LN (middle) and skin or tumor (right) sections from control mice (upper) and Amela-bearing mice (lower). The sections were stained for the proliferation marker Ki-67 (green), Gr1 (red) and the nuclei marker Topro3 (blue) as described (Soudja et al. 2010). (PDF) [file pone.0022639.s004.pdf]

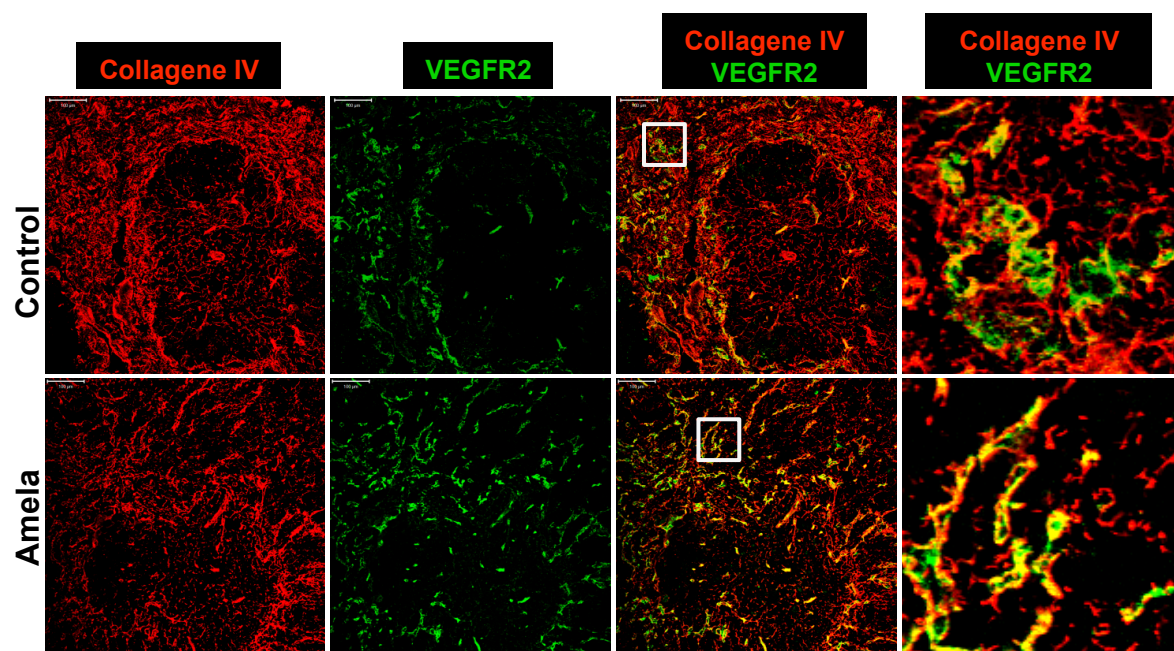

Supplemental figure 5

Supplement: Figure S5 — Stromal cells present in the splenic red pulp of Amela-bearing mice express the VEGFR2. Spleen sections from control and from Amela-bearing mice were stained with anti-collagen IV antibody (red) and with goat anti-mouseVEGFR2 (Flk-1) antibody from R&D Systems (green). Single stainings and merge images are shown. A magnification of the merged image is shown (far rigth). (PDF) [file pone.0022639.s005.pdf]

**A**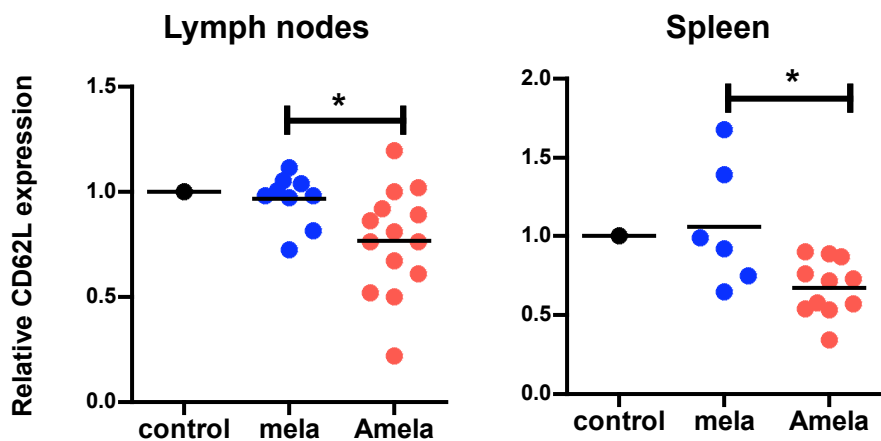**B**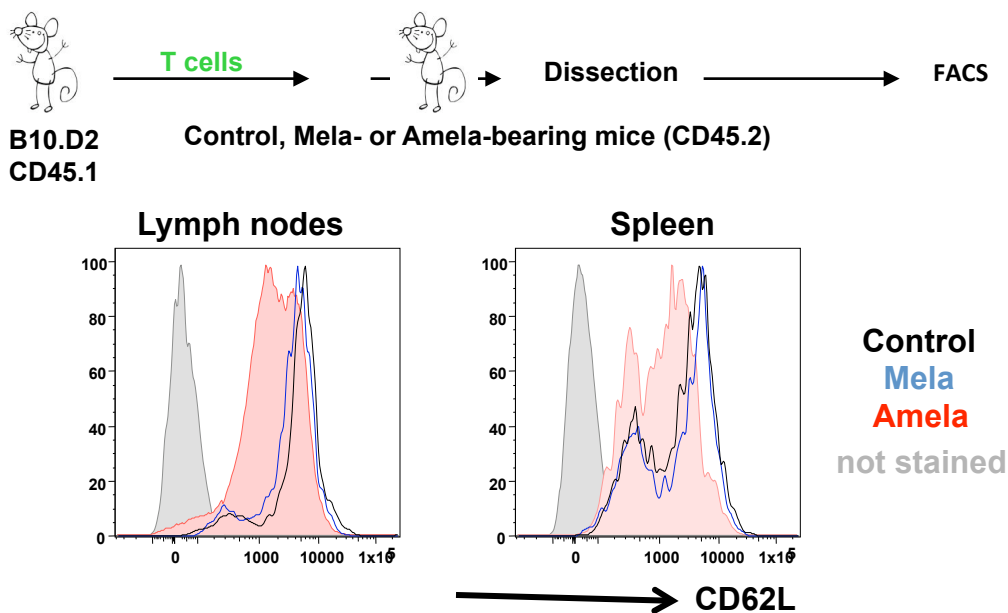**C**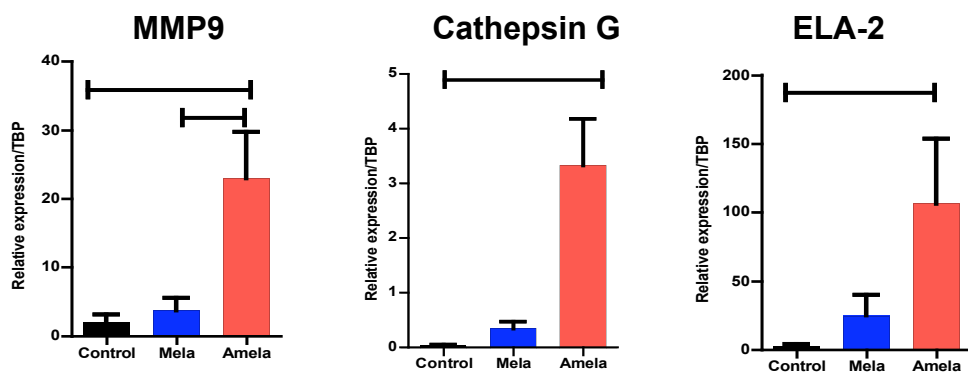

Supplement: Figure S6 — Potential mechanisms implicated in the disruption of SLO in Amela inflammatory tumor-bearing mice. (A) FACS analysis of CD62L expression on CD8 TL from LN and spleens of control mice (black), Mela- (blue), or Amela- (red) bearing mice. Mean fluorescence intensity values were normalized to those of the control samples. (B) TL from CD45.1 B10.D2 mice were purified and transferred in control mice, Mela- or Amela-bearing mice (all of which are CD45.2). 24 hrs later mice were sacrificed. FACS analysis of CD62L expression on transferred TL from control mice (black line), Mela- (blue line) or Amela- (red line) bearing mice is shown for LN (left) and spleen (right) samples. Gray lines are for unstained samples. (C) Quantitative RT-PCR for MMP9 (Mmp9), Cathepsin G (Ctsg) and Elastase-2 (ELA-2, Cela2a) transcript expression normalized to TBP (Tbp) mRNA expression in whole spleens from control, Mela- or Amela-bearing mice. (PDF) [file pone.0022639.s006.pdf]

**A**

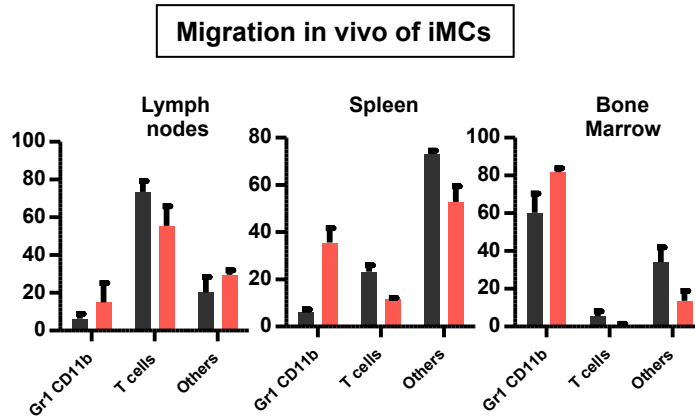

Recipient mice:  
Naïve  
Amela-bearing

**B**

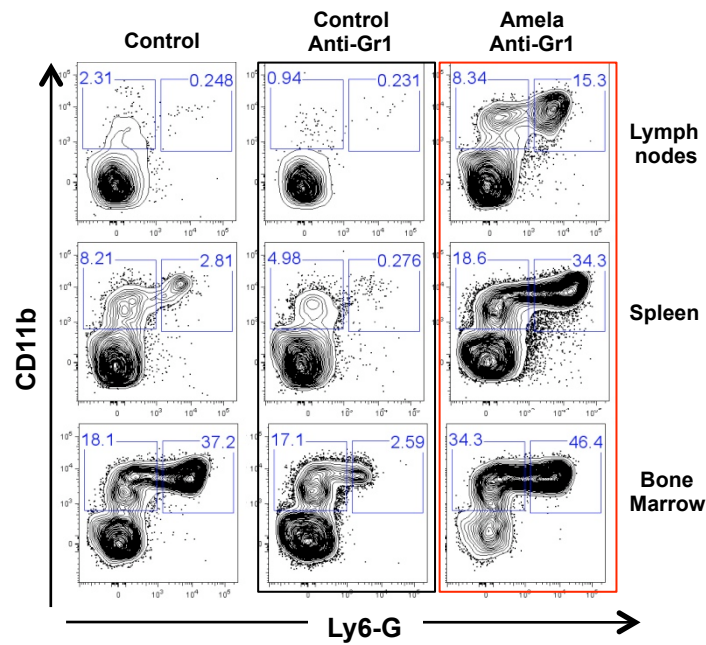

Treated mice:  
Naïve  
Amela-bearing

Supplemental figure 7

Supplement: Figure S7 — iMCs retrieved from spleens of Amela-bearing mice migrate to the bone marrow (BM) in tumor-free mice and anti-Gr1 mAb treatment fails to deplete them from Amela-bearing hosts. (A) Spleen cells from Amela-bearing mice rich in iMCs were labeled with CFSE 10 µM and transferred in tumor-free or in induced Amela-bearing mice. 20 hrs later LN, spleen and BM were harvested and FACS analysis was performed on CFSE+ gated cells. % of CFSE+ cells expressing Gr1 and CD11b (Gr1CD11b) or CD3 (T cells) is indicated as the mean +/− s.d. for 3 mice per group. Enrichment in Gr1+CD11b+ cells in spleens and in LN was observed only in Amela-tumor bearing mice. (B) Control and induced Amela-bearing mice were treated with 300 µg anti-Gr1 mAb (RB6-8C5) injected i.p. every 2 days for 6 days, were sacrificed at day 8 and LN, spleen and BM cells were analyzed by FACS for expression of CD11b and Ly-6G mAb (1A8), which recognizes an epitope of the Ly-6G/Gr1 molecule that is distinct from that recognized by mAb RB6-8C5. High percentages of CD11b+Ly6−G+ (iMCs) were still present in LN and spleen of Amela-bearing mice. Representative of two mice per group. (PDF) [file pone.0022639.s007.pdf]
