# Supplementary material for: Disrupted Lymph Node and Splenic Stroma in Mice with Induced Inflammatory Melanomas Is Associated with Impaired Recruitment of T and Dendritic Cells
Source: PLoS One. 2011 Jul 21;6(7):e22639. doi: 10.1371/journal.pone.0022639 (PMC3141075; doi:10.1371/journal.pone.0022639)
Supplement: Figure S8 — Examples of TiRP-10B Ink4a/Arfflox/flox B10.D2 mice with induced pigmented Mela- or amelanotic Amela-melanomas used in this study. (PDF) [file pone.0022639.s008.pdf]

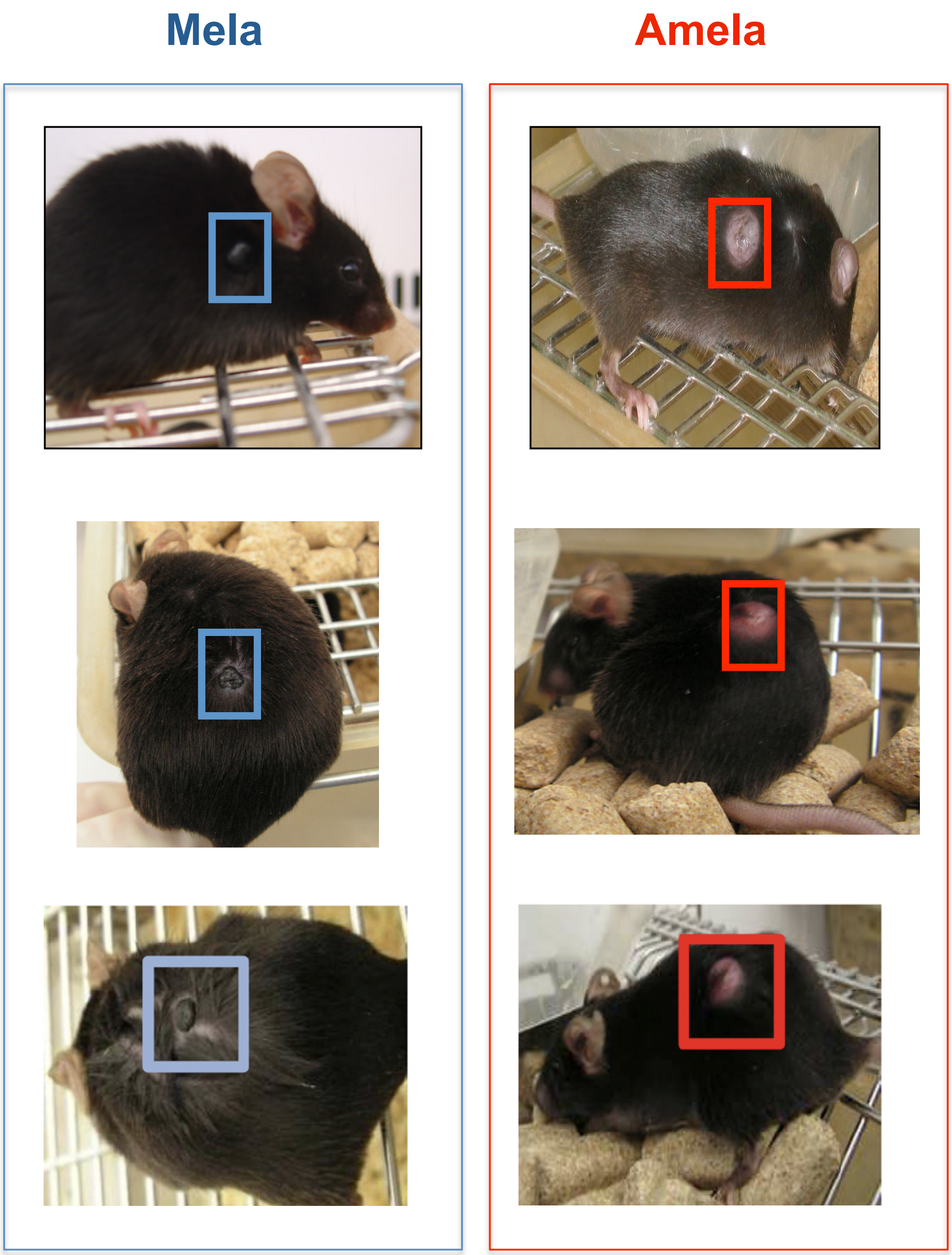

Supplementary Figure 8 : Examples of melanotic (Mela) and amelanotic (Amela) melanomas induced in TiRP Ink4aF/F B10.D2 mice after 4OH-tamoxifen treatment.
