# Supplementary material for: Disrupted Lymph Node and Splenic Stroma in Mice with Induced Inflammatory Melanomas Is Associated with Impaired Recruitment of T and Dendritic Cells
Source: PLoS One. 2011 Jul 21;6(7):e22639. doi: 10.1371/journal.pone.0022639 (PMC3141075; doi:10.1371/journal.pone.0022639)
Supplement: Table S1 — conserved numbers of TL in spleens of mice developing Amela-melanomas. (a) Spleen cells harvested from control, Mela- or Amela-bearing mice were counted and stained for CD45, CD4, CD8 and B220 expression by FACS as described (Soudja et al. 2010). N = number of mice analyzed. (b) % CD45+ cells among live spleen cells are given +/− SD. Corresponding cell numbers are given in bold. (c) % CD8 TL among CD45+ spleen cells +/− SD. Corresponding cell numbers are given in bold. (d) % CD4 TL among CD45+ spleen cells +/− SD. Corresponding cell numbers are given in bold. (e) % B220+ cells among CD45+ spleen cells +/− SD. Corresponding cell numbers are given in bold. (DOC) [file pone.0022639.s009.doc]

**Table S1: conserved numbers of TL in spleens of mice developing Amela-melanomas.**

| **Mice**^a^ | % CD45^+^ cells^b^  **nbr X 10^-6^** | % CD8^+^ TL^c^  **nbr X 10^-6^** | % CD4^+^ TL^d^  **nbr X 10^-6^** | % B220^+^ cells^e^  **nbr X 10^-6^** |
| --- | --- | --- | --- | --- |
| Control (N=68) | 81.6 ± 5.0 **65.3** | 10.8 ± 0.70  **7.1** | 15.8 ± 0.90 **10.3** | 50.4 ± 2.79  **32.9** |
| Mela (N=25) | 75.8 ± 8.9  **54.4** | 9.2 ± 0.72  **5.0** | 14.6 ± 2.04 **7.95** | 46.7 ± 4.26  **25.4** |
| Amela (N=50) | 45.1 ± 8.0 **210.4** | 5.3 ± 0.71  **11.0** | 7.2 ± 1.00 **15.14** | 25.6 ± 4.25  **53.9** |

1. Spleen cells harvested from control, Mela- or Amela-bearing mice were counted and stained for CD45, CD4, CD8 and B220 expression by FACS as described (Soudja et al. 2010). N= number of mice analyzed.
2. % CD45+ cells among live spleen cells are given +/- SD. Corresponding cell numbers are given in bold.
3. % CD8 TL among CD45+ spleen cells +/- SD. Corresponding cell numbers are given in bold.
4. % CD4 TL among CD45+ spleen cells +/- SD. Corresponding cell numbers are given in bold.
5. % B220^+^ cells among CD45+ spleen cells +/- SD. Corresponding cell numbers are given in bold.

**Table S2: murine primer sequences used in the real-time QRT-PCR amplifications shown in Fig.4B.**

| ***Gene*** (protein) | Forward | Reverse |
| --- | --- | --- |
| ***Ccl21a*** (Ccl21-ser) | ATCCCGGCAATCCTGTTCTC | GGTTCTGCACCCAGCCTTC |
| ***Ccl19*** (Ccl19) | CTGCCTCAGATTATCTGCCAT | AGGTAGCGGAAGGCTTTCAC |
| ***Cxcl13*** (Cxcl13) | GGAGTGATTTCAACTGTTGT | CATTTGGCACGAGGATTCACAC |
| ***Tbp*** (TATA box-binding protein) | CCTTCACCAATGACTCCTATGAC | CAAGTTTACAGCCAAGATTCAC |
