# Supplementary material for: Disrupted Lymph Node and Splenic Stroma in Mice with Induced Inflammatory Melanomas Is Associated with Impaired Recruitment of T and Dendritic Cells
Source: PLoS One. 2011 Jul 21;6(7):e22639. doi: 10.1371/journal.pone.0022639 (PMC3141075; doi:10.1371/journal.pone.0022639)
Supplement: Table S2 — Murine primer sequences used in the real-time QRT-PCR amplifications shown in Fig.4B and in Fig.S6C. (DOC) [file pone.0022639.s010.doc]

**Table S2: murine primer sequences used in the real-time QRT-PCR amplifications shown in Fig.4B and Fig.S6C.**

| ***Gene*** (protein) | Forward | Reverse |
| --- | --- | --- |
| ***Cela2a*** (Elastase-2) | AGGCATTGACTCCTTCATC | TTGATCCAATCTGCAAACTCAGC |
| ***Mmp9*** (MMP9) | GCA GAC CAA GAG GGT TTT CT | CTG GAA GAT GTC GTG TGA GTT |
| ***Ctsg*** (Cathepsin G) | GCCATCCTGATTACAATC | AGGCTTCACCGATCCACTCC |
| ***Ccl21a*** (Ccl21-ser) | ATCCCGGCAATCCTGTTCTC | GGTTCTGCACCCAGCCTTC |
| ***Ccl19*** (Ccl19) | CTGCCTCAGATTATCTGCCAT | AGGTAGCGGAAGGCTTTCAC |
| ***Cxcl13*** (Cxcl13) | GGAGTGATTTCAACTGTTGT | CATTTGGCACGAGGATTCACAC |
| ***Tbp*** (TATA box-binding protein) | CCTTCACCAATGACTCCTATGAC | CAAGTTTACAGCCAAGATTCAC |
