# Supplementary material for: Disrupted Lymph Node and Splenic Stroma in Mice with Induced Inflammatory Melanomas Is Associated with Impaired Recruitment of T and Dendritic Cells
Source: PLoS One. 2011 Jul 21;6(7):e22639. doi: 10.1371/journal.pone.0022639 (PMC3141075; doi:10.1371/journal.pone.0022639)
Supplement: Text S1 — Mice. On the B10.D2 (B10.D2/nOlaHsd, H-2d) background, the TiRP-10B Ink4a/Arfflox/flox B10.D2 mice housed in the CIML facility develop, after 4OH-tamoxifen treatment (injection s.c. twice two weeks apart of 4 mg 4OH-tamoxifen dissolved in ethanol and brought to 20 mg/ml with autoclaved sunflower oil as described (Huijbers et al. 2006)), single melanomas, either strongly pigmented (Mela) or unpigmented (Amela) with similar latency (on average 160 days) (Soudja et al. 2010). The incidence of Amela development was about 4 fold higher than that of Mela tumors. Mela- and Amela-tumor bearing mice analyzed in the present study each carried a single tumor, the size of the latter being on average twice that of the former. For examples, see Fig.S8. (DOCX) [file pone.0022639.s011.docx]

**Supplemental information:**

**Mice**:

On the B10.D2 (B10.D2/nOlaHsd, H-2^d^) background, the TiRP-10B Ink4a/Arf^flox/flox^ B10.D2 mice housed in the CIML facility develop, after 4OH-tamoxifen treatment (injection s.c. twice two weeks apart of 4 mg 4OH-tamoxifen dissolved in ethanol and brought to 20 mg/ml with autoclaved sunflower oil as described (Huijbers et al. 2006, ref. 16), single melanomas, either strongly pigmented (Mela) or unpigmented (Amela) with similar latency (on average 160 days) (Soudja et al. 2010, ref. 17). The incidence of Amela development was about 4 fold higher than that of Mela tumors. Mela- and Amela-tumor bearing mice analyzed in the present study each carried a single tumor, the size of the latter being on average twice that of the former. For examples, see Fig.S8.
